# Efficacy of Applying Hyaluronic Acid Gels in the Primary Prevention of Intrauterine Adhesion after Hysteroscopic Myomectomy: A Meta-Analysis of Randomized Controlled Trials

**DOI:** 10.3390/life10110285

**Published:** 2020-11-15

**Authors:** Min Cheng, Wen-Hsun Chang, Szu-Ting Yang, Hsin-Yi Huang, Kuan-Hao Tsui, Chia-Pei Chang, Wen-Ling Lee, Peng-Hui Wang

**Affiliations:** 1 Department of Obstetrics and Gynecology, Taipei Veterans General Hospital, Taipei 112, Taiwan; alchemist791025@gmail.com (M.C.); whchang@vghtpe.gov.tw (W.-H.C.); ss19910224@hotmail.com (S.-T.Y.); jpchang2@vghtpe.gov.tw (C.-P.C.); 2 Institute of Clinical Medicine, National Yang-Ming University, Taipei 112, Taiwan; khtsui60@gmail.com; 3 Department of Obstetrics and Gynecology, National Yang-Ming University, Taipei 112, Taiwan; 4 Department of Nursing, Taipei Veterans General Hospital, Taipei 112, Taiwan; 5 Biostatics Task Force, Taipei Veterans General Hospital, Taipei 112, Taiwan; sweethsin509@gmail.com; 6 Department of Obstetrics and Gynecology, Kaohsiung Veterans General Hospital, Kaohsiung 813, Taiwan; 7 Department of Pharmacy and Master Program, College of Pharmacy and Health Care, Tajen University, Pingtung County 907, Taiwan; 8 Department of Medicine, Cheng-Hsin General Hospital, Taipei 112, Taiwan; 9 Department of Nursing, Oriental Institute of Technology, New Taipei City 220, Taiwan; 10 Cancer Female Foundation, Taipei 104, Taiwan; 11 Department of Medical Research, China Medical University Hospital, Taichung 404, Taiwan

**Keywords:** anti-adhesive gel, hyaluronic acid, hysteroscopic myomectomy, intrauterine adhesion, prevention, reduction

## Abstract

Intrauterine adhesion (IUA), which mainly occurs after intrauterine surgery or an inflammatory process, is an important but often neglected condition in women of reproductive age. The presentation of IUA varies greatly, ranging from symptom-free to severe, with amenorrhea or infertility. With much advanced development of intrauterine instruments, more intrauterine diseases can be successfully cured by hysteroscopic surgery. Among these, submucosal myoma is one of the best examples. Submucosal myomas are often related to abnormal bleeding, anemia, and possible infertility or miscarriage. However, submucosal myoma after hysteroscopic myomectomy may be complicated by IUA in various grades of severity, and its incidence and prevalence might be nearly one-quarter to one-third of patients, suggesting an urgent need for efforts to decrease the risk of developing IUA after hysteroscopic myomectomy. Many strategies have been reported to be useful for this purpose, and intrauterine application of anti-adhesive gels, such as polyethylene oxide–sodium carboxymethylcellulose (PEO-NaCMC) or auto-crosslinked hyaluronic acid (ACHA), has become increasingly popular in routine clinical practice. This meta-analysis is aimed at investigating the effect of ACHA on the primary prevention of IUA formation after hysteroscopic myomectomy. A pooled analysis of three studies (hysteroscopic surgeries for fibroids, polyps, and septum) including 242 women showed that using PEO-NaCMC or ACHA gel decreased the IUA rate with an odds ratio (OR) of 0.364 (95% confidence interval (CI) 0.189–0.703, *p* = 0.03). Pooled analysis of two studies that limited the use of ACHA in 119 women showed that the application of ACHA gel for the primary prevention of IUA in patients after hysteroscopic myomectomy led to a statistically significant reduction of the development of IUA postoperatively (OR 0.285, 95% CI 0.116–0.701, *p* = 0.006). All of this suggests that the use of ACHA gel in patients after hysteroscopic myomectomy could significantly reduce de novo IUA, although more evidence is needed.

## 1. Introduction

Intrauterine adhesion (IUA) is a potentially chronic complication developed by the pathophysiology of trauma to the vascular basal layer of the endometrium, mainly as a result of hysteroscopic surgery, uterine curettage, termination of pregnancy, cesarean section, or genital tuberculosis or other severe inflammation processes [1,2,3,4,5,6,7,8,9]. IUA presents a challenge to the endometrial model of scar-free wound healing. In fact, the healing process of the endometrium is similar to the classical wound healing process, including three separate, continuous, and overlapping steps: hemostasis/inflammatory, proliferative, and remodeling phases [10,11,12,13,14,15]. In order to achieve scar-free regeneration and maintain endometrial integrity, at least three key components of endometrial biology should exist: (1) limited inflammation to prevent excessive tissue destruction, (2) cyclic activation of stem cells for regeneration, and (3) scar-free repair following menstrual shedding. Several postulated mechanisms for the loss of scar-free regeneration and repair have been proposed. They include hypoxic injury, unbalanced inflammatory process, decreased angiogenesis, disturbance of immune and molecular mechanisms, unregulated epithelial–mesenchymal transition, aberrant myofibroblast differentiation, bizarre stem cell regeneration, and interrupted normal endometrial cell proliferation [16,17]. IUA is a severe form of disruption of normal endometrial regeneration.

The basic histological finding of IUA is endometrial fibrosis. Avascular fibrous tissues and spindle-shaped myofibroblasts take the place of the originally normal stroma structure of the uterus [2,8]. Additionally, the normal endometrial glands are replaced by inactive cubo-columnar endometrial epithelium, which cannot be distinguished between stratum functionalis and stratum basalis [2,8]. Furthermore, this inactive single layer of cubo-columnar epithelium is almost completely nonresponsive to hormonal stimulation. Finally, fibrotic synechiae form across the entire uterine cavity, resulting in the most severe form of IUA, sometimes called Asherman syndrome [8,18,19,20,21,22,23]. According to Foix’s classification, three types of IUA have been proposed: (1) The most common type is in the form of avascular fibrous strands joining the uterine wall. In this type of IUA, thin-walled telangiectatic vessels can sometimes be found in the avascular fibrous strand. In addition, calcification and/or ossification can be found in the stroma area accompanied by spare and inactive or cystically dilated gland. (2) The second common type is muscular adhesion composed of collagen bundles, fibrous strips, or muscle with the same characteristics as normal myometrium, of which there is more than 50–80% of fibrous tissue in biopsy specimens. (3) The third type is sclerotic, atrophic endometrium [2,8,20]. Han and Du summarized the pathological changes of IUA, including endometrial fibrosis, endometrial scarring, loss or thinning of endometrium with different degrees of damage to the basal layer, atrophic gland, lack of vascular stromal tissue and hypoxia, and pale microenvironment in the adhesion area [22].

Women with IUA may present with various kinds of symptoms, and some are persistent. These symptoms include abnormal uterine bleeding, amenorrhea, dysmenorrhea, infertility, abnormal placentation, and recurrent miscarriage [1,2,4,7,8,9,24,25,26,27,28,29]. As there is continuous progression in hysteroscopic surgeries and they are widely performed for the treatment of various kinds of intrauterine lesions, there is increased concern about IUA-associated morbidities and the subsequent significant impairment of reproductive performance in women of reproductive age [3,5,7,30,31,32,33,34,35,36,37,38]. Among these surgeries, hysteroscopic myomectomy is one of the best examples, since it is considered as the best choice of therapy in the management of women with submucosal myomas [39,40,41,42,43,44,45,46]. However, the significantly increased risk of IUA after hysteroscopic myomectomy compared to other intrauterine surgeries, such as polypectomy, is well known [1,2,7,8,26,27,28,29,30,31,47,48,49,50,51].

Because of the wide variation of symptoms in women with the complication of IUA, late diagnosis is common. Some patients with IUA may have troublesome or even life-threatening clinical situations. These symptoms can be minimal but unpleasant, such as abnormal vaginal bleeding and/or intermittent vaginal spotting. Sometimes, symptoms can be severe, resulting in amenorrhea, and can be associated with pregnancy-related catastrophic diseases such as severe postpartum hemorrhage (PPH) and abnormal placentation, such as placenta accrete, increta, or percreta [52,53]. These IUA patients can be treated by hysteroscopic adhesiolysis after resolution of IUA and immediate restoration of the normal uterine cavity contour. However, hysteroscopic adhesiolysis is a relatively complicated surgery, associated with not only a high risk of surgery-related morbidity but also short-term therapeutic outcomes [1,2,7,8,26,27,37,54,55,56,57,58,59,60,61,62,63,64,65]. It is reported that in up to 62.5% of patients, IUA will recur after hysteroscopic adhesiolysis [54,55,56,57,58,59,60,61,62,63,64,65]. Taken together, this suggests the urgent need to focus on the primary prevention of IUA after intrauterine surgeries [3,5,7,30,31,32,33,34,35,36,37,38]. Several techniques have been proposed to prevent de novo IUA, which is postoperative adhesion without initial evidence of IUA at the same sites [66]. Physical barriers such as balloon catheters and intrauterine devices (IUD) have been used to decrease IUA after hysteroscopic surgery [24,26,30,31,35,38,46,58,59,60,61,64]. However, foreign body-related discomfort, inconvenience, increased infection rates, and possible uterine perforation are concerns [67]. In contrast, semi-solid agents can overcome the disadvantages of physical barriers. These materials include polyethylene oxide–sodium carboxymethylcellulose (PEO-NaCMC) gels and auto-crosslinked hyaluronic acid (ACHA) or hyaluronic acid (HA) gels, which have been proposed or investigated over the past few years [3,6,24,30,31,32,33,34,36,37,57,58,62,63,64,65,66,67]. ACHA and HA, in theory, show their effect on preventing the development of IUA based on their high affinity to the traumatic site of the postoperative endometrium [64]. However, a limited number of randomized controlled trials evaluating their efficacy in the primary prevention of IUA after hysteroscopic myomectomy have been conducted. The current meta-analysis was aimed at exploring the efficacy of ACHA and HA gels in hysteroscopic myomectomy for the primary prevention of de novo IUA.

## 2. Materials and Methods

### 2.1. Search Strategy and Study Selection

The meta-analysis was conducted based on the recommendation of Preferred Reporting Items for Systematic Reviews and Meta-analyses (PRISMA) and was registered in PROSPERO (ID: CRD42020176878) on 28/04/2020. We searched the PubMed, Embase, and ClinicalTrials.gov databases for relevant randomized controlled trials (RCTs) published online from their inception to May 2020. The search was performed without restrictions regarding language and country. Combined search terms included “hyaluronic acid”, “adhesion”, “intrauterine adhesion”, and “hysteroscopic surgery”. RCTs were eligible according to the following inclusion criteria: women undergoing hysteroscopic surgery for benign gynecologic disease, adhesion barrier of HA gel applied primarily at the end of surgery, and second-look hysteroscopy performed to identify the incidence and severity of IUA. Endpoints were reported as relative risk (RR) or odds ratio (OR) with corresponding 95% confidence interval (CI). Studies were excluded according to the following conditions: (1) patients with IUA before receiving surgery; (2) case reports, observational studies, or conference abstracts without adequate information for data synthesis; and (3) animal testing. Two reviewers (M.C. and P.-H.W.) independently evaluated all relevant articles retrieved from the databases according to the inclusion and exclusion criteria. Disagreements were resolved by discussion with the third author (W.-L.L.).

### 2.2. Procedures

Two investigators (M.C. and P.-H.W.) independently extracted data from each article, including authors’ names, publication year, study period, sample size, indication for surgery, type of hysteroscopic surgery, and incidence of primary IUA after surgery. Risk of bias was assessed by using the Cochrane Collaboration’s risk of bias tool covering allocation concealment, sequence generation, blinding, detection bias, attrition bias, and reporting bias.

### 2.3. Statistical Analysis and Data Synthesis

Heterogeneity between studies was evaluated using Cochran’s Q test and measured by I^2^ statistics. Low, moderate, and high heterogeneity were defined as I^2^ values of 25%, 50%, and 75%, respectively. A two-sided *p*-value of ≤0.05 was regarded as statistically significant. Comprehensive Meta-analysis Version 3.0 (Biostat Inc., Englewood, NJ, USA) was used for data synthesis [68]. A random effect model was used to calculate effect size in meta-analysis due to potential clinical heterogeneity from different surgical indications and investigated populations. Odds ratios (ORs) were calculated for dichotomous outcomes, with 95% CI measuring the effect of applying HA gels in hysteroscopic surgery versus no administration of anti-adhesion products according to the Cochrane Handbook for Systematic Reviews of Interventions Version 6, 2019.

## 3. Results

### 3.1. Strategy to Include Studies in the Current Meta-Analysis

**Figure 1:**
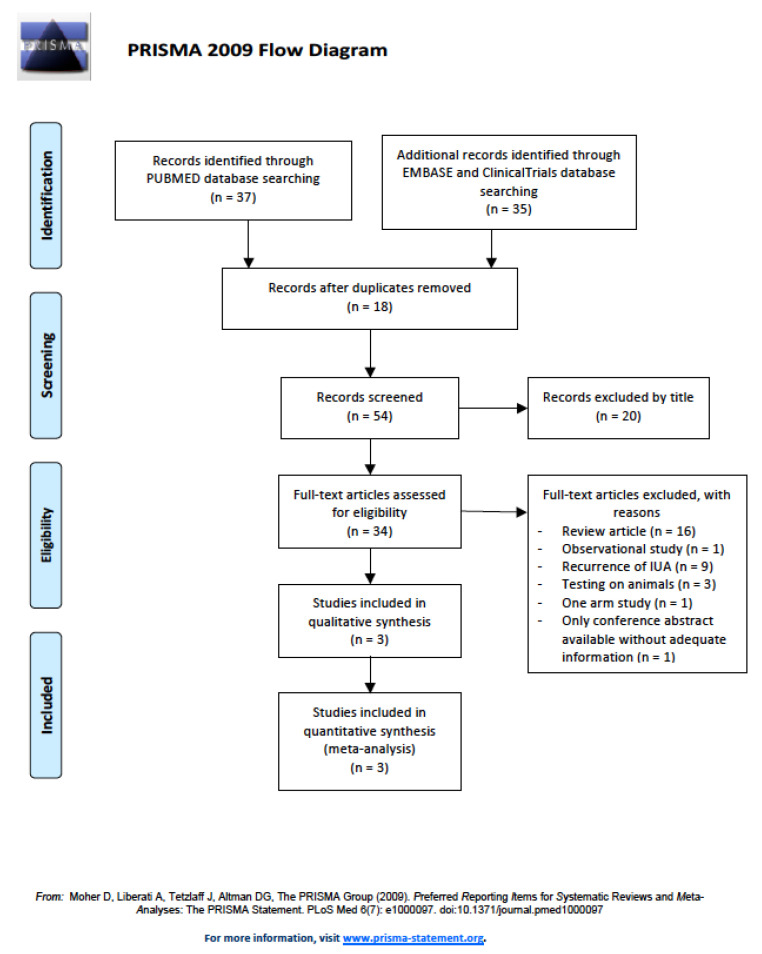
Preferred Reporting Items for Systematic Reviews and Meta-analyses (PRISMA) 2009 flowchart of studies in the current report.

After removing duplications and articles with unrelated topics, a total of 34 studies were reviewed in detail for eligibility; 31 studies were excluded, including 16 articles in review form, 1 observational study, 9 studies with evaluations of secondary intrauterine adhesion, 3 animal studies, 1 study with only one arm, and 1 conference abstract. Figure 1 shows the flowchart for identifying studies that met the criteria for the current meta-analysis. In the end, three randomized controlled studies [67,69,70] were included for meta-analysis.

### 3.2. Characteristics of Included Studies

The indications for hysteroscopic surgery were not consistent among the three studies [67,69,70]. Table 1 shows the basic characteristics of the included studies.

### 3.3. Quality of Included Studies

Table 2 shows an assessment of risk of bias, which was composed of five domains according to RoB 2, a revised Cochrane risk of bias tool for RCTs [71]. One study may have been at risk of randomization bias since it failed to report on allocation concealment [69]. Two studies failed to keep the investigation blind, which may have led to a higher risk of deviation from the intended intervention [67,70]. One study did not report the dropout rate [69], and the other two had dropout rates of 1.4% and 4.3% [67,70]. All dropout cases resulted from failing to attend follow-up hysteroscopy and outcomes were not evaluated based on intention to treat. However, dropout rates of the two studies were low, which may offset the risk of bias on missing data.

### 3.4. Effectiveness of Primary Prevention of Developing Intrauterine Adhesion in Patients Undergoing Hysteroscopic Surgery, Including Fibroid, Polyp, and Septum

On the evaluation of primary IUA rates, two of the three studies demonstrated a significant reduction (Table 3). The time of follow-up after operation ranged from 9 to 12 weeks. Guida et al. included cases of hysteroscopic surgery for myomectomy, polypectomy, and intrauterine septum resection, revealing 10.4% of IUA in the treatment group compared with 26.2% of IUA in the control group [67]. Huang et al. limited the patients with submucosal myoma treated by hysteroscopic myomectomy, and the results showed that 12.8% of patients had postoperative IUA in the treatment group compared with 39.1% in the control group [70]. On the other hand, De Iaco et al. did not show a significant difference in IUA rates between intervention and control groups, and their study also included different indications for hysteroscopic surgery, including myomectomy, polypectomy, and intrauterine septum resection [69].

**Figure 2:**
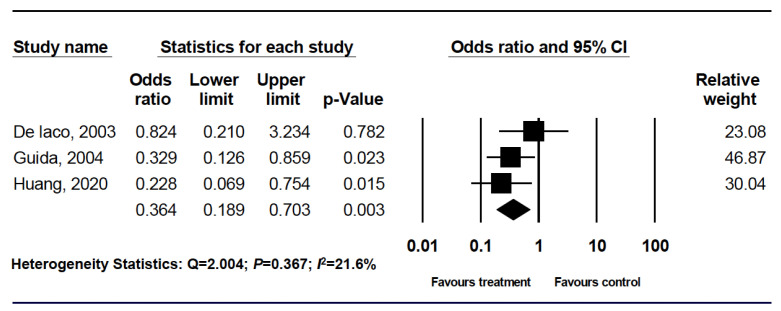
Forest plot comparing hyaluronic acid with control for intrauterine adhesion prevention.

All three of the analyzed studies included information on the IUA rate. For analysis, all three categories were included and pooled into the meta-analysis [67,69,70]. For these 242 patients, there was a significantly reduced risk of developing IUA in the ACHA and HA/NaCMC groups based on a random effect model (Figure 2).

### 3.5. Significant Reduction of Intrauterine Adhesion Rates in Patients Undergoing Hysteroscopic Myomectomy

While we focused on evaluating the effectiveness of applying ACHA in the primary prevention of IUA in patients after hysteroscopic myomectomy, two of the studies were included and pooled into the meta-analysis (Table 4) [67,71]. In Guida’s study, 49 patients were included in the analysis, contributing to an incidence of IUA of 16% in the ACHA treatment group and one-third in the group without ACHA [67]. Since all patients in Huang’s study were undergoing hysteroscopic myomectomy, upon further examination of their report, we found that two concentrations of ACHA (3% and 4%) were applied in the intervention group [70]. There was no statistically significant difference in the development of IUA between 3% and 4% ACHA application, although the trend showed a higher effect of 4% ACHA not only on the reduction of IUA incidence (17.4% vs. 8.3%, *p* = 0.352), but also on decreased severity (all had a mild degree of IUA in the 4% ACHA group and one-quarter had a moderate degree of IUA in the 3% ACHA group) [70]. However, compared with no use of ACHA in patients after hysteroscopic myomectomy, application of ACHA successfully decreased the incidence of IUA with both concentrations of ACHA gel (12.8% vs. 39.1%, *p* = 0.012) [70].

**Figure 3:**
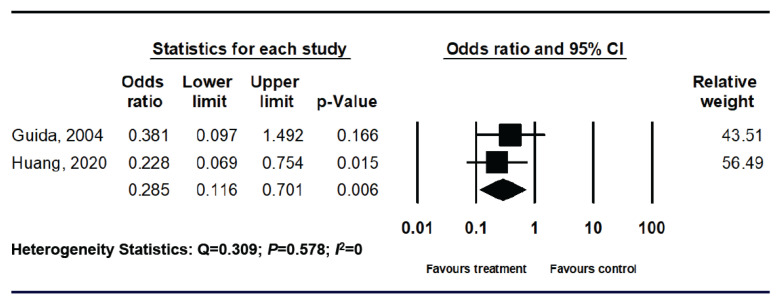
Forest plot comparing hyaluronic acid with control for intrauterine adhesion prevention in subgroup of hysteroscopic myomectomy.

For these 119 patients, there was a significantly reduced risk of developing IUA in the ACHA application groups based on the random effect model (Figure 3).

## 4. Discussion

The incidence of IUA after hysteroscopic surgery varies greatly depending on surgical indications and time of postoperative evaluation [1,2,3,4,5,6,7,8,9,24,26,30,47]. Taskin et al. reported IUA following hysteroscopic removal of a single myoma in 31.3% of cases, hysteroscopic resection for multiple myomas in 45.5% of cases, and hysteroscopic resection of intrauterine septum in 6.7% of cases [72]. On the other hand, a study by Yang et al. showed an incidence of IUA of 88% in patients who had undergone hysteroscopic septum resection and 40% in patients after hysteroscopic myomectomy, suggesting a significant proportion of IUA development after hysteroscopic surgery for more complicated diseases, such as uterine septation or myoma [35,36]. Although we found that a number of randomized controlled trials were performed to evaluate the application of ACHA gels as a barrier for the prevention of postoperative IUA, most of the studies did not exclude patients with IUA, and some studies also allowed adhesiolysis as one of the indications for hysteroscopic surgery, which could potentially show a relatively higher incidence of IUA resulting from intrauterine surgeries and underestimate the efficacy of ACHA gels on the primary prevention of IUA after hysteroscopic myomectomy. This meta-analysis focused on studies that enrolled patients who did not have IUA, with the expectation of conducting a more precise evaluation of the effect of ACHA gels on the primary prevention of IUA after hysteroscopic myomectomy.

Under similar clinical circumstances, including time of follow-up and surgical instruments used, Guida et al. [67] and Huang et al. [71] presented relatively consistent results on the efficacy of ACHA gels in the primary prevention of de novo IUA. Both studies revealed a significant reduction of the rate of IUA with the use of ACHA gels. This may be the first meta-analysis focusing on an evaluation of the incidence rate of de novo IUA with ACHA gels in patients who have undergone hysteroscopic myomectomy, demonstrating the low heterogeneity of eligible studies and a more conclusive effect with the use of a single anti-adhesion agent. However, the small sample size and limited number of available studies meeting our inclusion criteria were the major limitations of this meta-analysis, indicating an urgent need to include more randomized controlled trials to clarify the effect of ACHA as a tool for the primary prevention of IUA in patients following hysteroscopic myomectomy.

## 5. Conclusions

Applying ACHA gels in patients after hysteroscopic myomectomy could significantly reduce de novo IUA, although more evidence is needed.

## Figures and Tables

**Table 1:** Characteristics of included randomized controlled trials in the systematic review.

Study [Ref.]	n	Age (y)	Indication	Exclusion Criteria	Tools	Intervention	Follow-up Evaluation	DR
De Iaco, 2003 [69]	40	18–65	Fibroid Polyp Septum	Not stated	MR	HA/NaCMC, 10.5 ± 5.5 mL	9 weeks	Not stated
Guida, 2004 [67]	132	<50	Fibroid (n = 49) Polyp (n = 67) Septum (n = 16)	Postmenopause Pregnancy Prolapse Current illness Age > 50 y BW > 100 kg Other intrauterine lesions	BR	ACHA, 10 mL	3 months	4.3%
Huang, 2020 [70]	70	20–65	Fibroid (n = 70)	(1) Poor compliance with protocol (2) Known allergy to HA	BR	3 or 4% ACHA, 10 mL	12 weeks	1.4%

Fibroid, submucosal myoma; BW, body weight; MR, monopolar resectoscope; BR, bipolar resectoscope; HA, hyaluronic acid; NaCMC, sodium carboxymethylcellulose; ACHA, auto-crosslinked hyaluronic acid; DR, dropout rate.

**Table 2:** Risk of bias assessment of included studies.

Study [Ref.]	Bias Due to Randomization Process	Bias Due to Deviation from Intended Intervention	Bias Due to Missing Data	Bias Due to Outcome Measurement	Bias Due to Selection of Reported Results	Overall Risk of Bias
De Iaco, 2003 [69]	No information	No information	No information	No information	Some concerns	High
Guida, 2004 [67]	Low	Some concerns	Some concerns	Low	Low	Some concerns
Huang, 2020 [70]	Low	Low	Low	Low	Low	Low

**Table 3:** Summarized primary postoperative intrauterine adhesion rates of included studies.

Study [Reference]	Intervention		Control		*p*-Value
	n	Adhesion rate	n	Adhesion rate	
De Iaco, 2003 [69]	18	27.8%	22	31.8%	0.78
Guida, 2004 [67]	67	10.4%	65	26.2%	<0.05
Huang, 2020 [70]	47	12.8%	23	39.1%	0.012

**Table 4:** Summarized primary postoperative intrauterine adhesion rates of included studies (hysteroscopic myomectomy).

Study [Ref.]	Intervention		Control		*p*-Value
	n	Adhesion Rate	n	Adhesion Rate	
Guida, 2004 [67]	25	16.0%	24	33.3%	<0.05
Huang, 2020 [70]	47	12.8%	23	39.1%	0.012

## Abbreviations

The following abbreviations are used in this manuscript:

ACHA	auto-crosslinked hyaluronic acid
CI	confidence interval
D&C	dilation and curettage
IUA	intrauterine adhesion or intrauterine adhesions
NaCMC	sodium carboxymethylcellulose
OR	odds ratio
PEO	polyethylene oxide
PRISMA	Preferred Reporting Items for Systematic reviews and Meta-analyses
RCTs	randomized controlled trials
RR	relative risk
